# Using Web Search Query Data to Monitor Dengue Epidemics: A New Model for Neglected Tropical Disease Surveillance

**DOI:** 10.1371/journal.pntd.0001206

**Published:** 2011-05-31

**Authors:** Emily H. Chan, Vikram Sahai, Corrie Conrad, John S. Brownstein

**Affiliations:** 1 Children's Hospital Informatics Program, Harvard-Massachusetts Institute of Technology Division of Health Sciences and Technology, Boston, Massachusetts, United States of America; 2 Division of Emergency Medicine, Children's Hospital Boston, Boston, Massachusetts, United States of America; 3 Google Inc., Mountain View, California, United States of America; 4 Department of Pediatrics, Harvard Medical School, Boston, Massachusetts, United States of America; Yale School of Public Health, United States of America

## Abstract

**Background:**

A variety of obstacles including bureaucracy and lack of resources have interfered with timely detection and reporting of dengue cases in many endemic countries. Surveillance efforts have turned to modern data sources, such as Internet search queries, which have been shown to be effective for monitoring influenza-like illnesses. However, few have evaluated the utility of web search query data for other diseases, especially those of high morbidity and mortality or where a vaccine may not exist. In this study, we aimed to assess whether web search queries are a viable data source for the early detection and monitoring of dengue epidemics.

**Methodology/Principal Findings:**

Bolivia, Brazil, India, Indonesia and Singapore were chosen for analysis based on available data and adequate search volume. For each country, a univariate linear model was then built by fitting a time series of the fraction of Google search query volume for specific dengue-related queries from that country against a time series of official dengue case counts for a time-frame within 2003–2010. The specific combination of queries used was chosen to maximize model fit. Spurious spikes in the data were also removed prior to model fitting. The final models, fit using a training subset of the data, were cross-validated against both the overall dataset and a holdout subset of the data. All models were found to fit the data quite well, with validation correlations ranging from 0.82 to 0.99.

**Conclusions/Significance:**

Web search query data were found to be capable of tracking dengue activity in Bolivia, Brazil, India, Indonesia and Singapore. Whereas traditional dengue data from official sources are often not available until after some substantial delay, web search query data are available in near real-time. These data represent valuable complement to assist with traditional dengue surveillance.

## Author Summary

A variety of obstacles, including bureaucracy and lack of resources, delay detection and reporting of dengue and exist in many countries where the disease is a major public health threat. Surveillance efforts have turned to modern data sources such as Internet usage data. People often seek health-related information online and it has been found that the frequency of, for example, influenza-related web searches as a whole rises as the number of people sick with influenza rises. Tools have been developed to help track influenza epidemics by finding patterns in certain web search activity. However, few have evaluated whether this approach would also be effective for other diseases, especially those that affect many people, that have severe consequences, or for which there is no vaccine. In this study, we found that aggregated, anonymized Google search query data were also capable of tracking dengue activity in Bolivia, Brazil, India, Indonesia and Singapore. Whereas traditional dengue data from official sources are often not available until after a long delay, web search query data is available for analysis within a day. Therefore, because it could potentially provide earlier warnings, these data represent a valuable complement to traditional dengue surveillance.

## Introduction

With an estimated 500 million people infected each year [1], dengue ranks as one of the most significant mosquito-borne viral human diseases, and one of the most rapidly emerging vector-borne diseases [2], [3]. Considered to be endemic in over 100 countries, mostly in South-East Asia, the Americas and Western Pacific islands [3], recent estimates according to the Pediatric Dengue Vaccine Initiative put the population at risk, at 3.6 billion, or 55% of the world population.

Most national surveillance systems for dengue in endemic countries currently depend on passive or sentinel site surveillance of hospitalizations with some countries also monitoring outpatient clinics. However, weaknesses in these systems including non-streamlined bureaucratic structuring, politics and lack of funding for skilled personnel and equipment at local level laboratories have been cited as interfering with timely reporting and confirmation of cases [1], [4].

Alternative approaches to surveillance have turned to data outside of the virological or clinical domains with the hope of capturing health-seeking behavior at the earlier stages of disease progression, as well as capturing the population of the ill who do not seek medical care formally. Examples of these data include telephone triage calls [5], sales of over-the-counter drugs [6], school/work absenteeism [7], and online activity [8]–[12]. These data could complement traditional surveillance by potentially facilitating earlier detection, though results with respect to correlation and timeliness have been variable [13]. Even if the signals in one data source are no earlier than in another, there is benefit in using data that provide access to information on a more real-time or near real-time basis. The value of "predicting the present" for situations where data for the present may theoretically be available but not be accessible until the future is discussed in [14].

These novel approaches have so far for the most part been narrowly focused and validated on influenza-like and gastrointestinal illness. One example of such an effort is Google Flu Trends (http://www.google.org/flutrends/). The system mines Google search query data to estimate influenza activity in near real-time, developed by matching trends in queries for flu-related search terms to seasonal trends in the Centers for Disease Control and Prevention's (CDC) data for sentinel physician visits for influenza-like illnesses in the United States [9]. While the system has been successfully expanded to other nations to provide a near global picture of influenza activity, there is clearly value in applying these efforts to other pathogens where morbidity and mortality are more significant, where clinical outcomes are more severe or where a vaccine may not exist. Though one study has provided evidence for this broader potential application [15], in general few have evaluated the utility of web behavior data for other diseases and in non-English speaking countries.

In this paper, we describe the extension of the Google Flu Trends methodology to dengue surveillance. We provide initial results for Bolivia, Brazil, India, Indonesia, and Singapore and assess whether web search query data is a viable data source for the early detection and monitoring of dengue epidemics.

## Methods

### Overview

Our objective was to build models that are able to estimate a disease activity indicator for a significant high-burden disease by using data on Google search patterns. In building these models, time series of the fraction of Google search query volume for an appropriate disease from a particular country (both chosen by specific inclusion/exclusion criteria) were fit to a time series of case counts from official data sources. Our model fitting and query selection approach closely follows the precedent established by Google Flu Trends [9]. Statistical analyses were conducted using the statistical software R, version 2.10.1 (Vienna, Austria).

### Disease and Country Selection

Several factors had to be taken into consideration in selecting a specific disease and country around which a web query based surveillance tool would be developed. Such a tool would be most useful and successful for a high prevalence disease, in that the benefit of prevented cases gained by early detection would be maximized, but would work only where there is sufficient web searching behavior for information about the disease. However, concurrently, the disease should not be prone to “panic-induced searching” that would lead to spurious spikes in the data for our purposes. Our list of candidates was narrowed down further by the fact that model building would require a time series of official case counts against which web search query data could be fit and validated. Therefore, a further requirement was the availability of a corresponding official source dataset of case counts of at least a monthly but ideally weekly temporal resolution, dated 2003 or later (the time frame for which Google query data are available), and of at least three years in length. A final consideration was that the disease must exhibit fluctuations, via either a seasonal pattern or occasional upsurges, in order to better assess the match in trends in search data.

Initial disease and country candidates were identified based on considerations of annual national data reported by the World Health Organization (WHO) provided through its Global Health Atlas platform (http://apps.who.int/globalatlas/) to gauge the burden of a disease, and Google search query volume for queries about the disease in the country of interest to determine whether there was sufficient search interest. Attempts to find official case count data involved searching through various official websites including those of national Ministries of Health and the WHO, as well as scientific publications. We determined that endemic diseases were particularly suitable candidates because of their high burden, lesser susceptibility to panic-induced searching, and greater likelihood of available official data as they are often monitored by a national surveillance system. Ultimately, taking into consideration the above criteria, we decided to focus on dengue in Bolivia, Brazil, India, Indonesia, and Singapore.

### Data Sources

#### Google search query time series

By aggregating historical anonymized logs of online Google search queries submitted between 2003 and 2010, we computed time series of daily counts for the most common search queries in the selected countries, irrespective of query language. Separate daily counts were kept for every query in every country. Queries that occurred infrequently were excluded. Each time series was normalized by dividing the count for each query on a particular day by the total number of online search queries submitted in that country on that day, resulting in a search query fraction. Weekly and monthly fractions of these queries were also produced. No information about the identity of any user was retained, including IP address, once the country of origin was determined. Furthermore, any original web search logs older than nine months are made anonymous in accordance with Google's privacy policy (http://www.google.com/privacy/privacy-policy.html).

#### Official case count time series

A description of the official case count data used for each of the five chosen countries is provided in Table S1. All official data used were found online, from official websites of national Ministries of Health or the WHO, and are publicly available. These time series spanned a period during 2003-2010 and were of a weekly resolution for Bolivia and Singapore but monthly resolution for Brazil, India, and Indonesia. For India and Indonesia, actual case counts were not found, but approximate counts were reverse-extracted from official source graphs using GetData Graph Digitizer Version 2.24 (Moscow, Russia).

### Model Fitting

We fit a univariate linear model to the weekly or monthly official case count time series for each country independently:
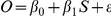
where *O* is the official dengue case count, *S* is the dengue-related Google search query fraction, *β_0_* is the intercept, *β_1_* is the multiplicative coefficient, and *ε* is the error term.

We split the official case count time series *O* into two sets: a training set *O_t_* and a holdout set *O_h_* of a complete outbreak season that spanned one year but not necessarily a calendar year. *O_h_* was used only for testing the final model. For *O_h_*, we generally selected the last full dengue season that was available in the time series; Singapore was the one exception because there had not been a significant outbreak that would be valuable to track from the point of view of the public health community since its 2007 season.

### Query Selection

Queries to include in the set of dengue-related Google search queries were selected separately for each country. This selection process started with first ranking individual search queries (from the total pool of all queries) according to their correlation with *O_t_*. Starting with the most correlated query, and discarding queries that were obviously unrelated to dengue, each query was sequentially added to the variable *S*. With each new addition, if the fit of the new model with *O_t_* improved, the query was kept in the set of queries, otherwise, the query was removed from the model and the query selection process was stopped at that point. We did not consider it necessary to utilize a cross-validation methodology during query selection given the small number of candidate queries that correlated well with *O_t_*, and given that we were estimating just two parameters, the intercept and the multiplicative coefficient. Therefore, model overfitting was not a major concern.

### Removal of Spurious Spikes

Spikes in the time series indicate an increase in interest in dengue, but it is important to determine whether they are “true spikes” representative of the burden of illness in the population or “spurious spikes” not reflective of population health impact. For example, spurious spikes may be caused by panic-induced searching when media attention about a particular outbreak triggers amplification of search activity that is disproportionate to the actual extent of the outbreak. These spurious spikes occur rarely but can be distinguished from true spikes when the rate of growth of the values in the time series *S* exceeds the normal rate of spread of the disease as determined by the basic reproduction number *R_0_*. In the absence of precise data about *R_0_*, we used a statistical approach to detect spurious spikes. Given a version of *S* based on daily data and a candidate spike point *p* that belongs to *S*, we computed the daily mean value and standard deviation using the previous four weeks' of daily data, and if *p* was found to exceed five standard deviations from the mean, we considered it to be a spike that was not driven by normal transmission of the disease. In such a case, we replaced *p* with a daily value imputed from the past data by simply continuing the trend of the last two weeks. We continued imputing subsequent points until the candidate points fell below the five standard deviation threshold. We chose such a high threshold to ensure that *S* will be modified only in extremely rare situations. Removal of spurious spikes was performed subsequent to query selection and prior to model fitting.

### Model Validation

Lastly, the predictive performance of the final model for each country was assessed by testing each model fit to the training set *O_t_* against the holdout set *O_h_* as well as the overall set *O*.

## Results

Dengue was determined to be a suitable candidate for search query-based disease surveillance since it generates over a million Google search queries every month. We found that the models contained up to ten queries for each country when built using the query selection approach described earlier. The queries were generally directly about dengue, expressed predominantly in Spanish, Portuguese, English, Indonesian and English for Bolivia, Brazil, India, Indonesia, and Singapore respectively. Some queries showed that the user was looking for more information about the disease, while others were looking for symptoms or treatments. Some of the queries contained misspellings of the word “dengue”. A few queries were related to mosquitoes and their control. There would be significant overlap between queries from different countries if translated to the same language. Training the models using different time periods of the truth data sometimes resulted in small changes to the list of selected queries, suggesting some elasticity in the query selection process.

**Figure 1:**
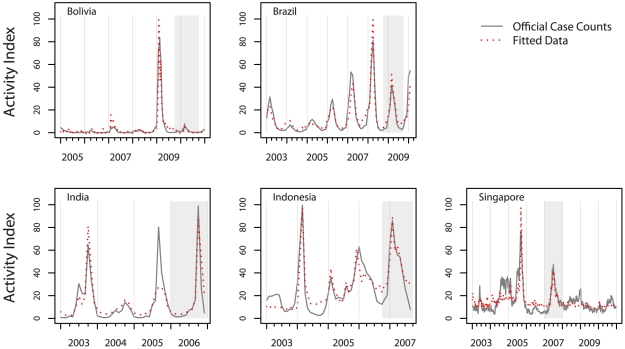
A comparison of the model-fitted and official case counts dengue epidemic curves in each country. The model-fitted epidemic curve as compared to the official case counts epidemic curve for dengue in each of the five countries for which a model built on Google search volume data was developed. Bolivia and Singapore are shown at a weekly resolution, the others on a monthly resolution. The activity index is a scaled measure of the case counts, representing the relative amount of dengue activity in each country on a scale from 0 to 100. Shaded regions indicate the season held out for testing the final models.

Model-fitted “expected” epidemic curves generally matched official case counts “observed” epidemic curves quite well for all five countries in most seasons, with the exception of Bolivia in 2007 when the model over-estimated the activity in that season, and India in 2005 for which it under-estimated (Figure 1). More formally, the correlation between values predicted by models fit to the training data and the holdout set as well as the overall dataset was generally quite high, ranging from 0.82 to 0.99 (Table 1).

**Table 1:** Model predictions were correlated with a holdout data subset and the overall dataset.

Country	Overall (*O*) Pearson's Correlation	Holdout (*O_h_*) Pearson's Correlation
Bolivia	0.94	0.83
Brazil	0.92	0.99
India	0.87	0.94
Indonesia	0.90	0.94
Singapore	0.82	0.94

## Discussion

Although there is a trend towards modernizing surveillance of infectious diseases, dengue surveillance is still very much traditional, mostly based on passive routine reporting or sentinel site surveillance, which is a preferable active but more costly approach [4]. The current standard approaches to dengue surveillance have recognized shortcomings including low sensitivity and accuracy and lack of timeliness. Therefore, the need to take steps to improve dengue surveillance has been well acknowledged [1], [4], [16], but cost and feasibility remain major obstacles.

The results of this study show that in general, models built on the fraction of Google search volume for dengue-related queries were able to adequately estimate true dengue activity according to official dengue case counts reported by national ministries of health or the WHO for five selected countries for the majority of the seasons during the time-frame analyzed. To our knowledge, few have explored non-traditional clinical/laboratory settings for monitoring dengue epidemics. Our results provide evidence of the availability of a novel data source that could supplement traditional surveillance. Furthermore, a web data based approach would be a low-cost option as it is passive and would require minimal resources to run.

The main added benefit in monitoring web-searching behavior is the potential for earlier detection. While notifications by doctors or laboratories to ministries of health are often delayed until there is a confirmed diagnosis [17], it is believed that individuals, especially at earlier stages of illness, may seek health information on the Internet before or even instead of making medical visits. One study evaluating a community-based surveillance system in rural Cambodia found that 67% of cases of hemorrhagic fever were treated at home as opposed to a health facility [18]. While rural areas are less likely to be served by Internet access, in other more developed areas, the Internet could be a source of information for those who do not actively seek clinical care. These data could therefore have the potential to provide earlier signals of epidemics in the community than clinical or laboratory data. Several studies have already demonstrated that web access logs and search query data work well for tracking influenza [9]–[12], although whether these data are actually timelier than traditional data is uncertain, with differing results depending on the study and gold standard of comparison.

However, even if the signals in web query data are no timelier than in traditional laboratory/clinical surveillance data, a tool built on the presented models could still provide a time advantage in that it would provide immediate access to an indicator of dengue activity that could help illustrate the dengue situation as it is currently. This idea reflects the concept of “now-casting” as opposed to forecasting, to predict the present rather than the future [14]. Official case counts are not always made publicly available in all countries, or if they are, there is a broad spectrum in the timeliness of when these data become available, ranging from only a couple days (as in the case of Singapore) to as much as months, or even years (as in case of the WHO's DengueNet system which collects data for all countries). This tool is not meant to serve to fill in these gaps with actual estimates of case counts, but by estimating an indicator of dengue activity that would be available in near real-time, it could serve as a stepping stone to prompt further investigation if warranted.

The lack of data stems from a variety of factors, including under-reporting. Even with mandatory reporting of dengue, under-reporting is prevalent [1], [4], [16]. Field investigations, sero-surveys and capture-recapture methods have yielded some remarkably low estimates for the sensitivity of dengue case notification, reflecting under-reporting [17], [19]–[21]. Reasons for under-reporting include lack of resources (both personnel and equipment), motivation and leadership, in addition to misunderstandings about or unfamiliarity with case definitions, complicated reporting procedures, a tendency to report only the most severe cases, lack of reporting from the private health sector [4], [16] and the reality that a proportion of the ill do not seek clinical care whether because they self-treat at home [18] or because their infection is asymptomatic or subclinical [21]. Unfortunately, the problem of under-reporting extends to our models as well as they were built on official data that are precisely affected by these problems. Therefore, it is not sensitivity but the ability to capture the same trends as the official data at a potentially earlier time point that is the value that a tool built on such models would be trying to capitalize.

A main challenge remains that rural areas and developing nations tend to lack or have limited Internet access currently. Web-query based surveillance depends on sufficient web search volume from any country of interest in order to both generate signals and drown out noise. In fact, it was this limitation of sufficient search volume that turned out to be a significant limiting factor in our process of identifying appropriate disease/location candidates.

Another limitation to be kept in mind with respect to expanding to different countries is that inter-country comparisons may be difficult due to differences in case definition for the official time series to which models were fitted. Unfortunately, because data using a consistent case definition across all countries do not exist (to our knowledge), each presented country and model must be considered independently.

Lack of Internet access may also be a potential explanation for the discrepancy between the fitted and actual values for the 2005 season in India. Though the gap is narrower today, there is a tremendous amount of regional variation in Internet penetration in India, a reflection of the country's economic disparity, especially between rural and urban areas [22]. The 2005 season was predominantly driven by a major outbreak that occurred in the state of West Bengal which includes the city of Kolkata, where per-capita Google searches at that time were much less than in cities like Delhi and Mumbai. Correspondingly, a model that fits aggregate national-level search data to national official case count data could underestimate true activity in regions with limited Internet usage. If state level data becomes available, future improvements to the model could include the addition of state-level adjustments.

Another limitation is that not everyone who submits a dengue related search query is actually ill with dengue. Indeed, a prevailing concern in such uses of web-searching behavior data for monitoring epidemic signals is the susceptibility of these data to panic-induced searching; the announcement of a novel outbreak, especially if compounded by media sensationalism, usually leads to increased online searching activity, and while a proportion of that behavior may be spurred by legitimate personal medical concern, a larger proportion is likely driven by fear or curiosity. By training the models over multiple years of data we are able to filter for terms that might be popular at a specific point in time during one season, but not over the multiple seasons. For example, when the first wave of H1N1 swine influenza emerged in 2009, there were large increases in search activity for “swine flu”, but this term was not included in Google Flu Trends models since it was not used significantly prior to 2009. Additionally, dengue is probably somewhat shielded from mass panic-induced searching; being an endemic disease in the regions we have focused on, dengue is less likely to receive the same degree of attention as would happen with a novel or rare disease. This hypothesis is confirmed by our results which demonstrate that dengue-related search queries are generally not as influenced by news coverage. For example, despite more severe and newsworthy outbreaks for Bolivia in 2009, Brazil in 2008, Indonesia in 2004, and Singapore in 2005, the models were able to handle these high levels of dengue activity without any significant overestimation. The one exception occurred in 2006 in India, when news about members of the prime minister's family being hospitalized for suspected dengue caused an unusually large spike in dengue queries. However, adjusting this spike prior to model fitting as described in our methods proved to be an effective way of retaining model fit. As with Google Flu Trends, despite strong historical correlations, our system remains susceptible to false alerts that could be caused by a sudden increase in dengue-related queries [9].

Incorrect self-diagnosis is another instance where dengue related search queries may not correspond to true illness. Notably, chikungunya and dengue are particularly difficult to distinguish because they manifest with similar symptoms and share the same vectors [23]. It has been made even more difficult since late 2005, when chikungunya re-emerged and led to a major outbreak in the Indian Ocean region, resulting in its current co-circulation with dengue in India [24]. Misdiagnosis is an obvious limitation for this tool, but it should be noted that in the case where it would be difficult for even doctors to make that distinction based on clinical symptoms alone, it is one that afflicts clinical data used in traditional surveillance of dengue as well. Therefore, misdiagnosis should be an acknowledged problem, but search query data could nonetheless be useful for evidence-based decisions, providing earlier signals on the basis of which more formal epidemiological investigation and coordination with diagnostic laboratories could be initiated.

Mining Google search query data raises obvious privacy concerns and it must be ensured that policies to protect personal information are extended to the application of this tool in public health practice. The main safeguard is that such a tool only presents query volume at the aggregate level where the unit of analysis prevents any re-identification of patients. The product of this work is freely available at www.google.org/denguetrends. The presented tool is not intended to replace traditional dengue surveillance, but by taking advantage of readily available data essentially provided by millions of individuals, it could be a useful and low-cost complement. These data can help mitigate some of the many gaps that exist in the current dengue surveillance landscape. More broadly, these results also contribute to a growing pool of evidence demonstrating the capability of relatively novel sources such as web-based data to assist with public health goals.

## Supporting Information

Table S1 Description and source of each official case count time series.
(DOC)
