# Supplementary material for: Using Web Search Query Data to Monitor Dengue Epidemics: A New Model for Neglected Tropical Disease Surveillance
Source: PLoS Negl Trop Dis. 2011 May 31;5(5):e1206. doi: 10.1371/journal.pntd.0001206 (PMC3104029; doi:10.1371/journal.pntd.0001206)
Supplement: Table S1 — Description and source of each official case count time series. (DOC) [file pntd.0001206.s001.doc]

**Supporting Information

Table S1. Description and source of each official case count time series.**

| Country | Dates | Temporal Resolution | Case Type* | Source | URL |
| --- | --- | --- | --- | --- | --- |
| Bolivia | 2005-2010 | Weekly | Suspected dengue fever cases | Ministerio de Salud y Deportes/Sistema Nacional de Información en Salud y Vigilancia Epidemiológica | http://www.sns.gob.bo/snis/default.aspx |
| Brazil | Jan. 2003-Apr. 2010 | Monthly | Reported dengue cases (excluding discarded) | Ministério da Saúde/SVS - Sistema de Informação de Agravos de Notificação - Sinan Net | http://dtr2004.saude.gov.br/sinanweb/tabnet/dh?sinannet/dengue/bases/denguebrnet.def (2007-2010) http://dtr2004.saude.gov.br/sinanweb/index.php (2003-2006) |
| India | 2003-2006 | Monthly | Reported dengue cases | WHO – South East Asia Regional Office | http://www.searo.who.int/LinkFiles/Dengue_dengue_india_seas_trend_03-06.pdf |
| Indonesia | Jan. 2003-Sept. 2007 | Monthly | Dengue cases | WHO – South East Asia Regional Office | http://www.searo.who.int/LinkFiles/Dengue_DengueSEAR-07.pdf (Page 2, Figure 1) |
| Singapore | 2003-2010 | Weekly | Dengue fever cases | Ministry of Health Singapore, Communicable Diseases Division | http://www.moh.gov.sg/mohcorp/statisticsweeklybulletins.aspx |

* Note: The case type given is in accordance with the term used by the data source. It was not always clear whether the case definition of, for example, “dengue cases” includes dengue hemorrhagic fever cases in addition to classical dengue fever cases.

WHO: World Health Organization
